# Cellular direct conversion by cell penetrable OCT4-30Kc19 protein and BMP4 growth factor

**DOI:** 10.1186/s40824-022-00280-8

**Published:** 2022-07-14

**Authors:** Seung Hyun L. Kim, Sungwoo Cho, Seoyeon Kim, Janet Kwon, Jaeyoung Lee, Rachel H. Koh, Ju Hyun Park, Hwajin Lee, Tai Hyun Park, Nathaniel S. Hwang

**Affiliations:** 1 Interdisciplinary Program in Bioengineering, Seoul National University, Seoul, 08826 Republic of Korea; 2 Department of Medicine, Standford University, 450 Serra Mall, Standford, 94305 USA; 3 School of Chemical and Biological Engineering, Institute of Chemical Processes, Seoul National University, Seoul, 08826 Republic of Korea; 4 Department of Biomedical Engineering, University of California, Davis, CA 95616 USA; 5 Department of Biomedical Science, Kangwon National University, Gangwon-do, Chuncheon, 24321 Republic of Korea; 6 Max/N-Bio Institute, Institute of Bioengineering, Seoul National University, Seoul, 08826 Republic of Korea; 7 School of Dentistry, Seoul National University, Seoul, 08826 Republic of Korea; 8 Uppthera, BRC Laboratory, Yeonsu-gu, Incheon, 21990 Republic of Korea

**Keywords:** Transdifferentiation, Osteogenesis, Angiogenesis, HUVECs, Cell penetrating protein

## Abstract

**Background:**

The number of patients suffering from osteoporosis is increasing as the elderly population increases. The demand for investigating bone regeneration strategies naturally arises. One of the approaches to induce bone regeneration is somatic cell transdifferentiation. Among the transcriptional regulators for transdifferentiation, octamer-binding transcription factor 4 (OCT4) is famous for its role in the regulation of pluripotency of stem cells. Bone morphogenetic protein 4 (BMP4) is another factor that is known to have a significant role in osteogenic differentiation. Previous studies have achieved transdifferentiation of cells into osteoblasts using viral and plasmid deliveries of these factors. Although these methods are efficient, viral and plasmid transfection have safety issues such as permanent gene incorporations and bacterial DNA insertions. Herein, we developed a cell penetrating protein-based strategy to induce transdifferentiation of endothelial cells into osteoblasts via nuclear delivery of OCT4 recombinant protein combined with the BMP4 treatment. For the nuclear delivery of OCT4 protein, we fused the protein with 30Kc19, a cell-penetrating and protein stabilizing protein derived from a silkworm hemolymph of Bombyx mori with low cytotoxic properties. This study proposes a promising cell-based therapy without any safety issues that existing transdifferentiation approaches had.

**Methods:**

OCT4-30Kc19 protein with high penetrating activities and stability was synthesized for a protein-based osteogenic transdifferentiation system. Cells were treated with OCT4-30Kc19 and BMP4 to evaluate their cellular penetrating activity, cytotoxicity, osteogenic and angiogenic potentials in vitro. The osteogenic potential of 3D cell spheroids was also analyzed. In addition, in vivo cell delivery into subcutaneous tissue and cranial defect model was performed.

**Results:**

OCT4-30Kc19 protein was produced in a soluble and stable form. OCT4-30Kc19 efficiently penetrated cells and were localized in intracellular compartments and the nucleus. Cells delivered with OCT4-30Kc19 protein combined with BMP4 showed increased osteogenesis, both in 2D and 3D culture, and showed increased angiogenesis capacity in vitro. Results from in vivo subcutaneous tissue delivery of cell-seeded scaffolds confirmed enhanced osteogenic properties of transdifferentiated HUVECs via treatment with both OCT4-30Kc19 and BMP4. In addition, in vivo mouse cranial defect experiment demonstrated successful bone regeneration of HUVECs pretreated with both OCT4-30Kc19 and BMP4.

**Conclusions:**

Using a protein-based transdifferentiation method allows an alternative approach without utilizing any genetic modification strategies, thus providing a possibility for safer use of cell-based therapies in clinical applications.

**Supplementary Information:**

The online version contains supplementary material available at 10.1186/s40824-022-00280-8.

## Introduction

As the elderly population increases, the number of people who suffer from osteoporosis escalates due to the aging skeleton losing bone volume and mass. Osteoporosis results from an imbalance in catabolic and anabolic activities by osteoclasts and osteoblasts [1–3]. Several drugs are approved related to osteoporosis, such as bisphosphonates, Denosumab, Romosozumab, etc. However, their ability to derive bone regeneration process is severely limited. One of the alternative approaches which could directly induce bone regeneration is cell-based therapy. Since imbalance in bone metabolism often arises from functional and genetic damage in osteoblasts, the generation of new functional osteoblasts is a key to restoring normal bone [1, 4]. Various types of stem cells are often osteogenically differentiated as an alternative cell source of osteoblasts [5–10]. Among these, induced pluripotent stem cells (iPSCs) were derived to become osteogenic cells [5–8]. However, the current trend in cell reprogramming discourages the use of iPSCs due to the possible arouse of genomic aberrations and risk of oncogenesis [11–13]. Thus, somatic cell transdifferentiation without such limitations is preferred.

Somatic cell transdifferentiation has been applied in various cell types such as neuronal cells, hepatocytes, endothelial cells, skeletal myocytes, chondrocytes, pancreatic cells, osteoblasts, etc. [14–20]. To obtain osteoblasts, previous studies have used various combinations of transcription factors for transdifferentiation [20–23]. Among these, incorporating osteogenic transcriptional factors, RUNX2 and OSTERIX, and stem cell-specific transcriptional factors, OCT4 and L-MYC, to human dermal fibroblasts demonstrated successful derivation of osteoblastic cells by Yamamoto et al. [20]. In the same study, introducing octamer-binding transcription factor 4 (OCT4) alone induced elevated alkaline phosphatase (*ALP*) and osteocalcin (*OCN*) RNA expressions and positive von Kossa staining in fibroblasts which elicit a significant role of OCT4 in osteogenic transdifferentiation. The importance of OCT4 in inducing transdifferentiation has been observed in other cases as well [24–26]. In addition to OCT4, bone morphogenetic protein 4 (BMP4) is another essential factor in osteogenesis [27, 28]. It is known as a key regulator of skeletal tissue formation in the developmental process via activating the SMAD pathway [29, 30]. The role of BMP4 is not limited to osteogenesis but expands to reprogramming cells such as promoting pluripotency in the early reprogramming phase of iPSC generation from fibroblasts and eliciting endothelial-mesenchymal transition in endothelial cells [31, 32]. Thus, both OCT4 and BMP4 are crucial regulators in osteogenesis.

Previous studies have achieved transdifferentiation of cells into osteoblasts using viral and plasmid deliveries of multiple transcription factors or treating chemicals and/or inhibitors [20–23]. Although these methods demonstrated efficient osteogenic conversion, viral and plasmid transfections cause safety risks such as permanent gene incorporations and bacterial DNA insertions [33]. In addition, both viral and plasmid constructs are in DNA forms resulting in cells to process through mRNA transcription and protein translation. Cell-penetrating transcription factor delivery in protein form can serve as a replacement for the above methods without eliciting safety risks. Some cell-penetrating peptides include forms derived from α-helix of Antennapedia homeodomain protein, trans-activating regulatory protein, TAT, from HIV, VP22 protein from herpes virus, and poly-arginine peptide sequence [34–38]. The cell-penetrating peptide, Pep-c19, allows the protein and its cargo protein to penetrate the cell membrane [39]. Cell-penetrating protein 30Kc19 has been previously studied for cell-penetrating capability, soluble protein expression, and protein stability of cargo proteins [40–43]. A previous study has shown efficient soluble protein production and cellular delivery of OCT4, SOX2, and KLF4 by fusion of these transcription factors with 30Kc19 [43]. Cell-penetrated transcription factors remained stable within cells until 48 h. In this study, we showed OCT4 fused with 30Kc19 protein, OCT4-30Kc19, and BMP4 treatment elicited osteogenic as well as angiogenic differentiation in human umbilical vein endothelial cells (HUVECs) in vitro, in 3D cell spheroids, and in vivo.

## Materials and methods

### Cell culture

Human umbilical vein endothelial cells (HUVECs, Lonza, C2519A) were cultured in EGM-2 medium. Cells were maintained at 37 ℃ with 5% CO_2_. During OCT4-30Kc19 and BMP4 growth factor treatment, cells were maintained in EBM-2 with 1% FBS without growth factor supplements from EGM-2 kit (Lonza). Osteogenic differentiation was carried out in StemPro™ Osteogenesis medium (Thermo Fisher) and the medium was changed every two to three days.

### Plasmid construction and protein purification

The *OCT4-30Kc19* plasmid used in the previous study was used [43]. OCT4-30Kc19 proteins were collected via fast protein liquid chromatography (FPLC, GE Healthcare) with elution buffer (20 mM Tris–HCl, 0.5 M NaCl, 350 mM imidazole, pH 8.0), and the solvent was changed to Endothelial cell growth basal medium-2 (EGM-2, Lonza).

### Coomassie blue staining and western blot analysis

To confirm the size and presence of OCT4-30Kc19 protein, the purified product from FPLC was run in 7.5% sodium dodecyl sulfate–polyacrylamide (SDS-PAGE) gel electrophoresis. The purified protein was denatured by 10 min of boiling in the 5X sample buffer (LPS Solution). For coomassie blue staining, the SDS-PAGE gel was put in coomassie blue staining solution (0.1% Brilliant blue R [Merck] in 40% ethanol 10% acetic acid) for 2 h followed by de-staining (40% ethanol 10% acetic acid) for 2 h. For western blot analysis, SDS-PAGE gel was transferred onto polyvinylidene difluoride (PVDF) membrane via iBlot kit (Thermo Fisher). The protein-containing membrane was blocked with 3% bovine serum albumin (BSA) (MP Biomedicals) 0.1% Tween-20 (Merck) in tris-buffered saline (TBS) (Bio-rad) for 1 h. Then, the sample was incubated in 1% BSA/TBS containing OCT4 primary antibody (1:1000, Abcam, 19,857) overnight. After incubation in secondary antibody (1:2000) for 1 h, Clarity Western ECL Substrate (Bio-rad) was used as HRP substrate. For visual imaging of the band, the G: BOX Chemi XL system (Syngene) was used.

### Recombinant protein and growth factor treatment

HUVECs were cultured in endothelial serum-free media without any supplements, such as growth factors and hormones. After serum starvation with EBM-2 for 24 h, cells were maintained in EGM-2 with 1% FBS without growth factor supplements from the EGM-2 kit. Cells were treated with 10 ng/ml of BMP4 and 40 μg/ml of OCT4-30Kc19 for 48 h, and then the media was changed into the osteogenesis media.

### Cell cytotoxicity

Cell cytotoxicity was measured with a LIVE/DEAD® cell viability kit (Thermo Fisher). Ethidium homodimer-1 and calcein AM stained dead and live cells, respectively. HUVEC cells in 2D or 3D cell spheroid were incubated with the mixed solution of Ethidium homodimer-1 and calcein AM at 37 ºC in a humidified CO_2_ incubator for 5 min or 30 min, respectively. For visual imaging, confocal laser scanning microscopy (Carl Zeiss) was used. The cytotoxicity of MNPs in HUVECs was examined by WST-8 Cell Counting Kit (Dojindo), following the manufacturer’s instructions. In brief, after the incubation of HUVECs with various concentrations of MNPs in 96-well plates at 37 °C in a humidified CO_2_ incubator for 1,3, and 7 days, cells were incubated with cell culture medium supplemented with 10% WST-8 solution for an additional 2 h in the incubator. The absorbance of each group was measured at 450 nm by a microplate reader (Tecan Infinite m200).

### Magnetic nanoparticles preparation

Magnetic nanoparticles (MNPs) were prepared as previously described [44, 45]. Briefly, *Magnetospirillium* sp. strain AMB-1 was cultured in magnetic spirillum growth media (ATCC) for 2 weeks under fed-batch conditions. After ultrasonic disruption of bacterial cells, MNPs were isolated and purified. Followed by sterilization and quantification with ICP-AES (ICPS-7500, Shimadzu), 1 mg/ml of MNPs were dispersed in phosphate-buffered saline (PBS) buffer.

### HUVEC spheroid generation

3D cell spheroids were generated as previously described [45, 46]. Briefly, when the HUVECs reached 70–80% confluency, 20 μg/ml of MNPs were incorporated into the cells by incubation with MNP-mixed EGM-2 medium for 24 h. Then, only the magnetized cells were sorted by using NdFeB magnets and seeded into the magnetic pin-array platform. The number of HUVECs was 100,000 cells/well. After inducing transdifferentiation, the morphology of the spheroid was observed using optical microscopy (Olympus).

### Tube formation assay

Cells from each group were collected and seeded on Matrigel (BD Biosciences) in 24-well. After 6 h of incubation in 2% FBS EGM-2 medium containing vascular endothelial growth factor (VEGF, Lonza), tube formations were observed. Visualization of cell morphology or tube formation was carried out with rhodamine-phalloidin (Invitrogen) actin staining. Fixated cells were incubated in rhodamine-phalloidin actin staining solution for 2 h and were ready for imaging.

### Alizarin red s staining

After fixation with 4% PFA, cells were stained with alizarin red s staining solution (Sciencell) for 30 min to 1 h. The remaining staining solution not bound to calcium was washed away with deionized water (DW). 10% acetic acid was added to stained samples after washing. Then, samples were collected for heating at 85 ℃ for 10 min. Heated samples were centrifuged and 10% ammonium hydroxide was added to the supernatant to neutralize the acid. The absorbance of the final product was measured at 405 nm. For 3D spheroid cells, the cell spheroids were chopped into pieces and then attached to the bottom of the well plates by centrifugation. Then, alizarin red s staining was conducted with gentle pipetting.

### RNA isolation, cDNA generation, and quantitative real-time PCR

PCR samples were collected via Trizol® reagent (Thermo Fisher) treatment on cells. Following Trizol treatment, chloroform was added and samples were incubated at room temperature for 10 min. Then, samples were separated into two layers by centrifugation at 15,000 rpm for 20 min at 4 ℃. A clear layer containing RNA was collected and isopropanol was added for precipitation. After another centrifugation at 15,000 rpm for 20 min at 4 ℃, the RNA pellet was washed in 75% ethanol followed by centrifugation at 10,000 rpm for 10 min at 4 ℃. The collected RNA pellet was denatured in molecular biology water (Merck) at 60 ℃ for 10 min. cDNA samples were generated from isolated RNA via reverse-transcriptional PCR with cDNA kit (Enzynomics, EZ006M) following the manufacturer’s protocol. cDNA concentration was measured with Infinite® 200 PRO (Tecan). 100 ng of cDNA was used for quantitative RT-PCR analysis with SYBR green PCR Mastermix via StepOnePlus™ Real-Time PCR System (Applied Biosystems).

### Cryogel fabrication

Gelatin-heparin cryogel was previously studied and used in different studies [25, 47]. 1% (w/v) Type A porcine gelatin (Sigma) and 0.3% (w/v) heparin (Merck) were dissolved in DW. 1-ethyl-3-(3-(dimethylamino)propyl)carbodiimide (EDC) (Thermo Fisher) and sulfo-hydroxysuccinimide (NHS) (Thermo Fisher) were used to crosslink gelatin and heparin. After mixing solutions, 200 μl of the solution was loaded onto pre-sterilized scaffold molds. Then, samples were incubated at -20 ℃ overnight.

### Surgical protocol for subcutaneous delivery and cranial mouse models

Animal experiments followed the Guide for the Care and Use of Laboratory Animals by the Seoul National University (SNU-170728–1-5). Balb/c-nude mice were maintained in climate-controlled rooms at 22 ℃ with 50% humidity and 12 h light/dark cycles. Mice were anesthetized with Alfaxan® (Jurox) and Rompun® (Bayer) to minimize the suffering and stress during surgical procedures. For cell delivery into the subcutaneous tissue, a longitudinal incision was made on the back of mice and cell-seeded scaffolds were delivered. Samples were collected after 6 weeks. Mice with cranial defects were made with a 4 mm diameter dental drill at the center of the sagittal crest on the head. Cell-seeded scaffolds were put in the defect area and were collected after 8 weeks for further bone regeneration.

### Histological analysis

Collected samples were embedded in paraffin and were cut in 4 μm thickness. Sectioned slides were put under xylene, 100%, 95%, 90%, 80%, ethanol, and water for deparaffination and were subjected for Hematoxylin & Eosin, Masson’s trichrome, or protein immunofluorescent staining.

### Immunostaining

Four percent paraformaldehyde (PFA, Merck) was used to fix cells for 20 min at room temperature. Fixated cells were put in permeabilization solution containing 0.2% Triton-X100 (Merck) for 15 min. Blocking solution consisted of 10% normal goat serum (NGS, Vector Laboratories) was added for 1 h. Cells were incubated in primary antibody solution containing OCT4 (1:400, Abcam, ab19857), CD31 (1:200, BioLegend, 303,110), αSMA (1:200, Abcam, ab5694), or OCN (1:400, Santa Cruz, sc-74495), or TIE-2 (1:400, Santa Cruz, sc-293414) antibody in 0.1% Triton-X100, 5% NGS overnight. Cells were washed for secondary antibody against mouse (1:400, Thermo Fisher, A21151) or rabbit (1:400, Thermo Fisher, A11008) for 1 h. Nuclei were stained with DAPI (1:200, Merck, D9541) for 15 min. For immunostaining protocol on histological samples, antigen was retrieved with proteinase K (1:100, Merck) solution before permeabilization. Visual imaging was carried out with confocal laser scanning microscopy, Eclipse Ti2 inverted-microscope (Nikon), or super-resolution microscope (SRM, Carl Zeiss). Image analysis such as merging RFP, GFP, and DAPI channels or measuring mean fluorescence intensity was carried out with ImageJ (NIH).

### Micro-computed tomography

Regenerated bone volume quantification and images of new bone formation in the defect area were acquired via Skyscan 1171 (SkyScan) at 59 kV, 167 μA, and 40 ms of exposure. CT images were taken every 1° with a full rotation of 360°. Collected images were reconstructed and quantified with ReCon Micro-CT software.

### Statistical analysis

Standard deviation (SD) is represented in all data. Significant differences were analyzed by one-way ANOVA followed by Tukey’s post hoc test. Significance is shown in data with p values less than 0.05: ^*^p < 0.05, ^**^p < 0.01, ^***^p < 0.001.

## Results

### Intracellular and nuclear delivery of OCT4-30Kc19

**Fig. 1:**
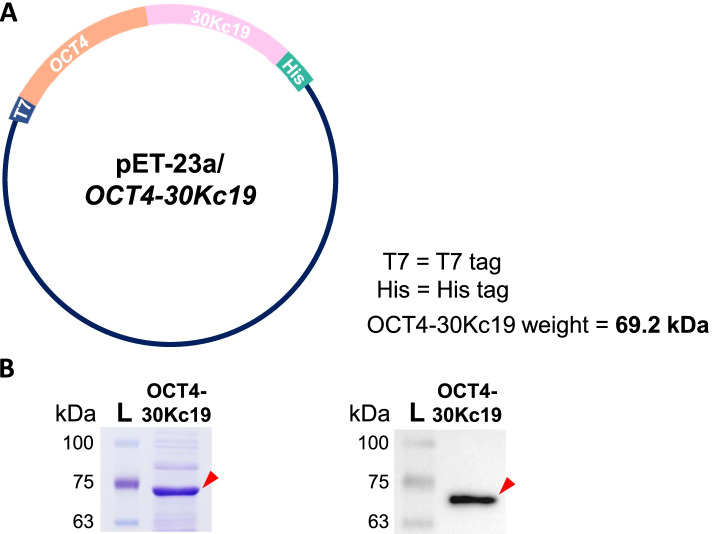
Plasmid map and confirmation of OCT4-30Kc19 cell-penetrating protein production. (**A**) Plasmid construction of pET-23a/OCT4-30Kc19 that is 69.2 kDa in size. T7, T7 tag. His, His tag. (**B**) Confirmation of OCT4-30Kc19 protein using coomassie blue staining and western blot analysis. Locations of band sizes, which are about 69.2 kDa were consistent in both coomassie blue staining and western blot analysis (marked with red arrowhead). Anti-OCT4-antibody (1:1000) was used for OCT4-30Kc19 detection in western blot analysis. L, protein ladder

**Fig. 2:**
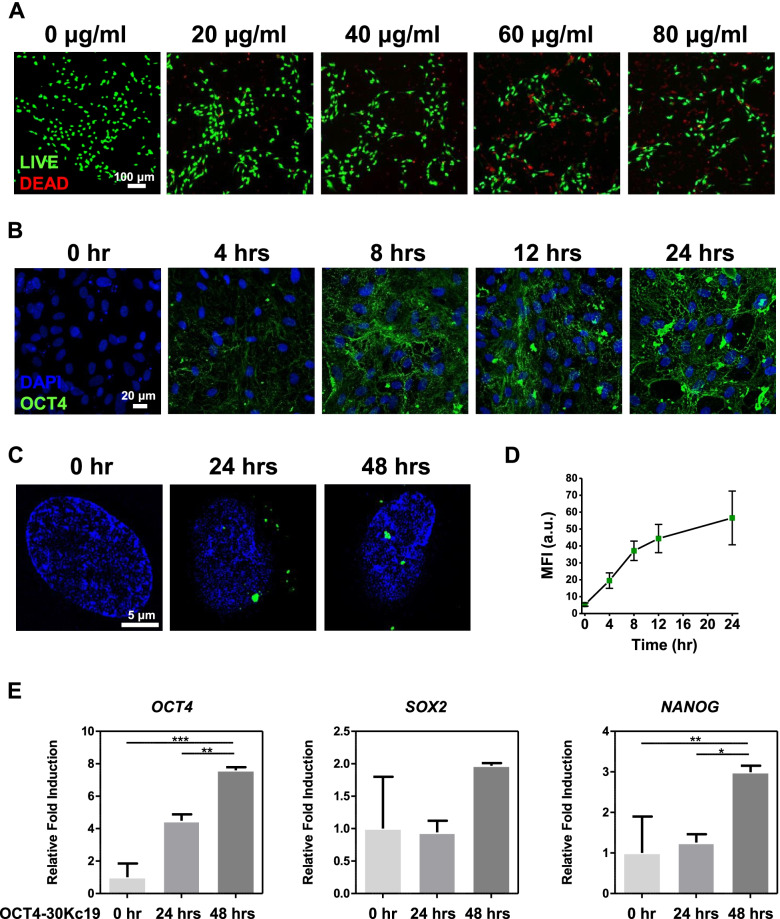
Cytotoxicity and cell penetration measurement on HUVECs. (**A**) Representative images of live and dead cells. After treating HUVECs with different concentrations of OCT4-30Kc19 protein for 24 h, cells were stained by live and dead kit. Scale bar, 100 μm. HUVECs treated with 20 and 40 μg/ml of OCT4-30Kc19 showed similar high viability, while higher concentrations lowered viability. Accordingly, 40 μg/ml of OCT4-30Kc19 was used throughout the experiment. (**B**) Cellular penetration of OCT4-30Kc19 protein after 0, 4, 8, 12, 24 h of a protein treatment. When cells were treated with OCT4-30Kc19, protein effectively penetrated HUVECs from 0 to 24 h. Anti-OCT4-antibody (1:400) was used for OCT4-30Kc19 detection. Blue, green represent the nucleus and OCT4-30Kc19 protein, respectively. Scale bar, 20 μm. (**C**) Nucleus penetration of OCT4-30Kc19 protein after 0, 24, 48 h of a protein treatment. OCT4-30Kc19 protein was localized in the nucleus and acted as a transcription factor for pluripotent genes in 24 and 48 h samples. Blue, green represent the nucleus and OCT4-30Kc19 protein, respectively. Scale bar, 5 μm. (**D**) Nucleus penetration of OCT4-30Kc19 protein in a time-dependent manner. OCT4-30Kc19 protein increased over time. Quantitative analysis of fluorescence was performed using image J software (*n* = 20). (**E**) Quantitative polymerase chain reaction (qPCR) for pluripotency transcription factors, *OCT4, SOX2, NANOG*. HUVECs were treated with OCT4-30Kc19 for 24 and 48 h, and then PCR analysis was conducted. Gene expression levels of pluripotency transcription factors were normalized to *GAPDH*. PCR analysis showed elevation of *OCT4, SOX2, NANOG* expression as OCT4-30Kc19 treatment time increased (*n* = 3). ^*^p < 0.05, ^**^p < 0.01, ^***^p < 0.001

Cell penetrating protein OCT4 fused with 30Kc19 was previously studied for inducing the soluble expression and enhancing the stability of OCT4 recombinant protein [43]. The plasmid structure of *OCT4-30Kc19* is shown in Fig. 1A. Confirmation of OCT4-30Kc19 protein production was seen in coomassie blue staining and western blot analysis of the final product (Fig. 1B). Locations of band size, which is about 69.2 kDa, were consistent in both coomassie staining and western blot analysis (marked with red arrowhead). For our transdifferentiation study, we utilized HUVECs to induce conversion into osteogenic cells. To optimize the protein concentration for minimal toxicity, OCT4-30Kc19 protein concentration was treated on HUVECs for cytotoxicity measurement. HUVECs were treated with OCT4-30Kc19 in varying concentrations of 0, 20, 40, 60, and 80 μg/ml. Live/dead analysis of HUVECs after 24 h of protein treatment showed increasing cytotoxicity as concentration elevated (Fig. 2A). HUVECs treated with 20 and 40 μg/ml of OCT4-30Kc19 showed similar live cell percentages of 89 and 85%. Protein concentration at 60 and 80 μg/ml significantly lowered percentages of live cells to 65 and 61% (Supplementary Fig. 1). Thus, the maximum amount of protein that causes the lowest cytotoxicity, 40 μg/ml, was used throughout the experiments. After optimizing protein concentration, intracellular and nuclear delivery of OCT4-30Kc19 was examined. As hours increased from 0 to 24 h, intracellular delivery of the protein was readily visible (Fig. 2B). In addition to intracellular delivery, nuclear delivery of OCT4-30Kc19 was confirmed in 24 h and 48 h samples (Fig. 2C). OCT4-30Kc19 proteins seem to be localized more in the nucleus in a time-dependent manner (Fig. 2D). This was confirmed through PCR analysis in which *OCT4, SOX2,* and *NANOG* showed elevation in fold induction by 7.6-, 1.97- and 2.99-folds, respectively (Fig. 2E).

### Cellular response after OCT4-30Kc19 and BMP4 treatment on HUVECs

**Fig. 3:**
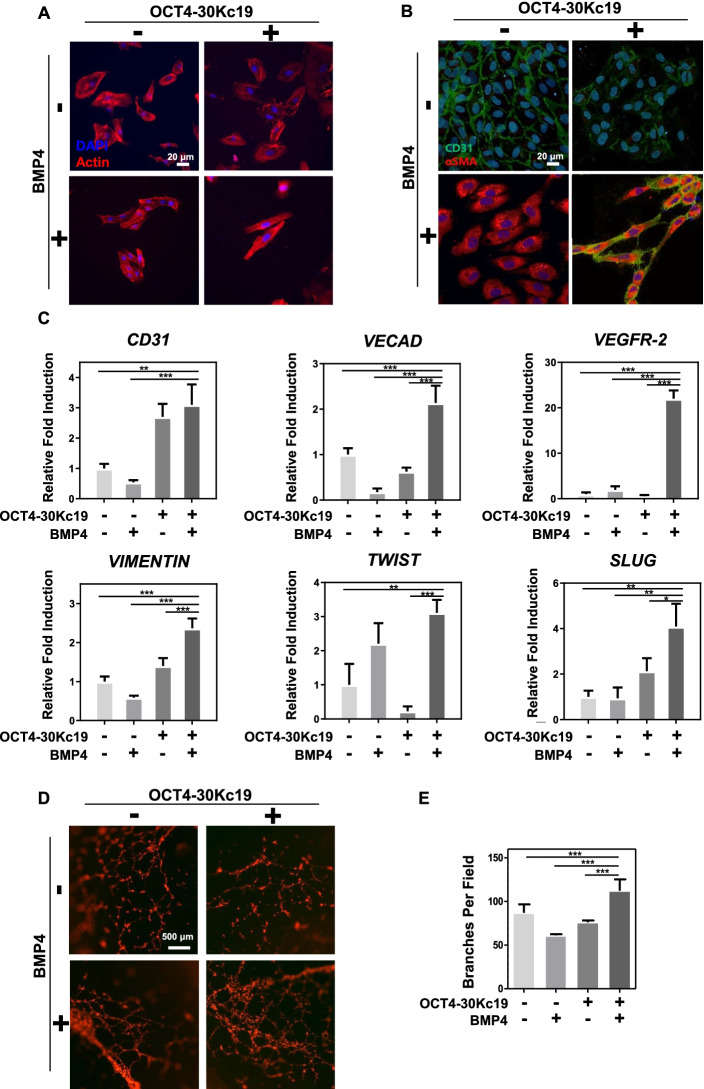
Morphological, phenotypical, and genomic changes in HUVECs after OCT4-30Kc19 and BMP4 treatment. (**A**) Morphological changes in HUVECs after OCT4-30Kc19 and BMP4 treatment. Stimulation of HUVECs with 40 μg/ml of OCT4-30Kc19 and 10 ng/ml of BMP4 for 48 h resulted in elongated morphology in HUVECs. Cell morphology was visualized with rhodamine-phalloidin staining. Blue, red represent the nucleus and actin structure, respectively. Scale bar, 20 μm. (**B**) Phenotypical changes in HUVECs after OCT4-30Kc19 and BMP4 treatment. Immunofluorescence images of protein-treated HUVECs showed elevated expression of CD31 and αSMA. Anti-CD31-antibody (1:200) and anti-αSMA-antibody (1:200) were used as primary antibodies. Blue, green, red represent the nucleus, CD31, and αSMA, respectively. Scale bar, 20 μm. (**C**) Genomic changes in HUVECs after OCT4-30Kc19 and BMP4 treatment. Quantitative polymerase chain reaction (qPCR) analysis for endothelial, mesenchymal, and endothelial-mesenchymal transition (EndMT) markers showed an elevation in fold induction after both protein treatments (*n* = 3). ^*^p < 0.05, ^**^p < 0.01, ^***^p < 0.001. (**D**) Tube formation assay to evaluate the effects of OCT4-30Kc19 and BMP4 on angiogenesis. Tube formation of HUVECs was visualized with phalloidin staining after OCT4-30Kc19 and BMP4 treatment. Scale bar, 500 μm. (**E**) Quantitative analysis of branches per field (*n* = 3). ^***^p < 0.001

In addition to OCT4-30Kc19, BMP4 proteins were treated for 48 h on HUVECs to induce further osteogenic conversion. Treatment of both OCT4-30Kc19 and BMP4 resulted in elongated morphology in HUVECs (Fig. 3A). HUVECs undergo morphological changes such as branching and elongation, which can be interpreted as an initial and crucial behavior in the process of establishing microvascular networks [48]. Endothelial and mesenchymal marker immunostaining showed a phenotypical change in HUVECs treated with OCT4-30Kc19 and BMP4 (Fig. 3B). Both CD31 and αSMA expression were prominent in OCT4-30Kc19 and BMP4 treated HUVECs. Confirmation of phenotypical change with PCR gene expression showed the addition of OCT4-30Kc19 and BMP4 elevated expressions in endothelial, mesenchymal, and endothelial-mesenchymal transition (EndMT) markers (Fig. 3C). Specifically, increased *CD31, VECAD*, and *VEGFR-2* expressions in OCT4-30Kc19 and BMP4 treated group was 3.1-, 2.1-, and 22.02-folds, respectively. The largest increase in *VEGFR-2* suggested angiogenic capacity in OCT4-30Kc19 and BMP4 treated HUVECs. This was confirmed via tube formation assay in which cells treated with OCT4-30Kc19 and BMP4 elicited higher branch numbers (Fig. 3D and E). In addition, elevated gene expressions of mesenchymal and EndMT markers, *VIMENTIN, TWIST,* and *SLUG*, by 2.3-, 3.1- and 4.09-folds, respectively, demonstrated mesenchymal characteristics in OCT4-30Kc19 and BMP4 treated HUVECs. Treatment of both OCT4-30Kc19 and BMP4 elicited morphological, phenotypical, and genomic changes in HUVECs and induced both angiogenic and mesenchymal characteristics. This data conforms with previously presented results in which *OCT4* plasmid DNA transfection followed by BMP4 growth factor treatment eliciting dual characteristics in HUVECs [25].

### Increased osteogenic differentiation capacity of 2D-cultured HUVECs after protein treatment

**Fig. 4:**
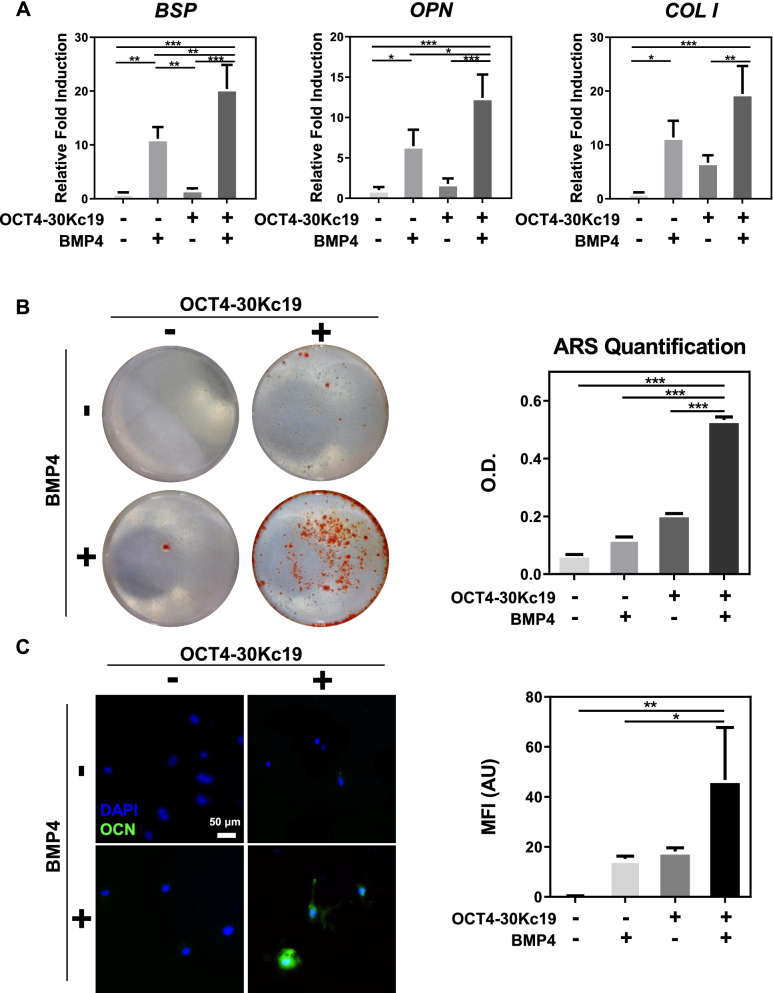
In vitro osteogenic differentiation in HUVECs after OCT4-30Kc19 and BMP4 treatment. (**A**) Quantitative polymerase chain reaction (qPCR) for osteogenic marker genes, *COL I, OPN*, and *BSP*. HUVECs were treated with OCT4-30Kc19 and BMP4 for 2 weeks in osteogenesis medium. PCR analysis showed elevation in osteogenic markers when HUVECs were treated with OCT4-30Kc19 and BMP4 (*n* = 3). ^*^p < 0.05, ^**^p < 0.01, ^***^p < 0.001. (**B**) Alizarin red s staining (ARS) showing calcium deposition in HUVECs. The sample treated with OCT4-30Kc19 and BMP4 showed the highest calcium deposition. Quantitative analysis of ARS (*n* = 3). ^***^p < 0.001. (**C**) Immunofluorescence staining of OCN in HUVECs. OCN was expressed in HUVECs treated with OCT4-30Kc19 and BMP4. Anti-OCN-antibody (1:400) was used as the primary antibody for OCN detection. Blue and green represent the nucleus and OCN, respectively. Scale bar, 50 μm. Mean fluorescence intensity (MFI) of each group was quantified with Image J software (*n* = 3). ^*^p < 0.05, ^**^p < 0.01

To confirm osteogenic capacity, cells from each group were cultured in osteogenic medium for two weeks. After two weeks of osteogenic culture, HUVECs treated with OCT4-30Kc19 and BMP4 showed a significant increase in *COLI, OPN,* and *BSP,* osteogenic gene markers, by 19.49-, 12.43- and 20.39-folds, respectively (Fig. 4A). Following PCR data, alizarin red s staining showed the highest calcium deposition in HUVECs treated with OCT4-30Kc19 and BMP4 (Fig. 4B). Quantification of staining confirms the accumulated calcium content. Additional immunofluorescence staining demonstrated OCN presence in HUVECs treated with OCT4-30Kc19 and BMP4 (Fig. 4C). Mean fluorescence intensity measurement showed elevated intensity in the same group. Together, osteogenic gene expression profile, presence of osteogenic protein and high calcium deposition capability confirm cell conversion into functional osteoblastic cells in vitro.

### Increased osteogenic differentiation capacity of 3D HUVEC spheroids after protein treatment

**Fig. 5:**
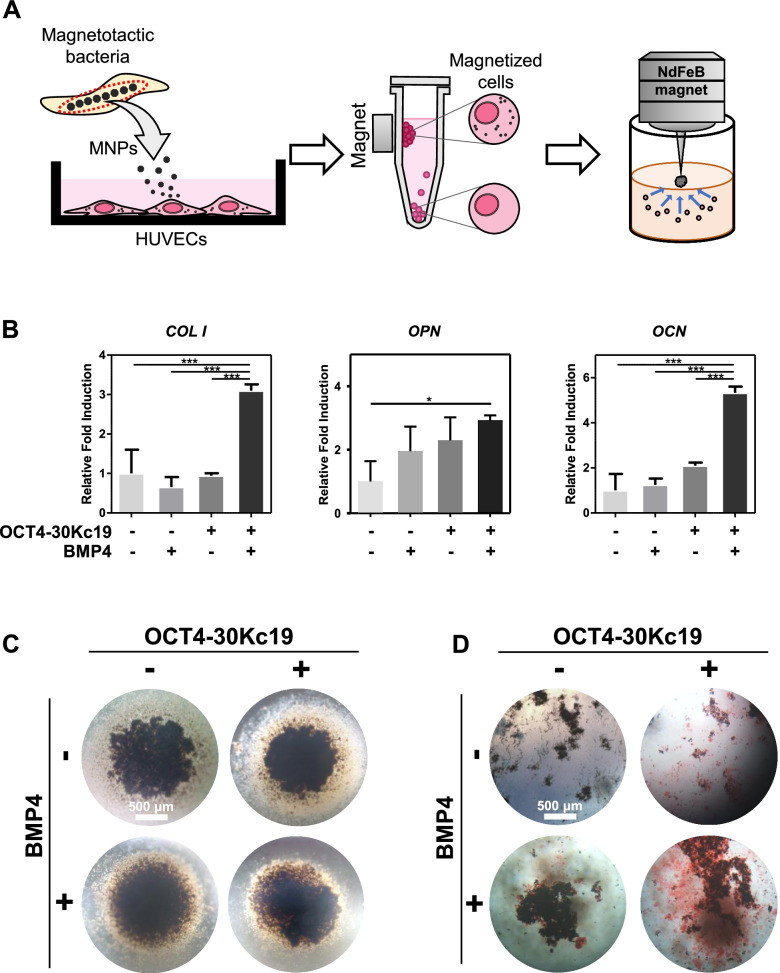
Osteogenic differentiation of HUVEC spheroids after OCT4-30Kc19 and BMP4 treatment. (**A**) Schematics of the HUVEC spheroids generation procedures. After incubation with MNPs mixed medium, HUVEC were detached and then magnetized HUVECs were sorted by using static magnets. Then, MNP-incorporated HUVECs were seeded into the magnetic pin array platform to generate 3D HUVEC spheroids. (**B**) Quantitative polymerase chain reaction (qPCR) for osteogenic marker genes, *COL I*, *OPN*, and *OCN*. HUVEC spheroids were treated with OCT4-30Kc19 and BMP4 for 2 weeks in osteogenesis medium. PCR analysis showed elevation in osteogenic markers when HUVECs were treated with OCT4-30Kc19 and BMP4 (*n* = 3). ^*^p < 0.05, ^***^p < 0.001. (**C**) Morphology of the 3D cell spheroids after 2 weeks of osteogenic induction. The number of initial cell seeding was 100,000 cells per well of the magnetic pin-array platform, yielding HUVEC spheroids of around 1,000 μm in diameter. The size of the spheroids was remaining the same without a loss for 2 weeks of osteogenic induction. Scale bar, 200 μm. (**D**) Alizarin red s staining (ARS) showing calcium deposition in HUVEC spheroids. The sample treated both with OCT4-30Kc19 and BMP4 showed the highest calcium deposition

After 24 h of incubation with MNP-mixed media, sufficiently magnetized HUVECs were sorted by applying magnetic force using magnets to the cell suspension. Only the magnetized HUVECs were then seeded into 96-well plates with the magnetic pin-array platform to generate HUVEC spheroids (Fig. 5A). The concentration of MNPs, 20 μg/ml, for magnetization of HUVEC cells was selected as the maximum amount that showed no cytotoxicity (Supplementary Fig. 2). HUVEC spheroids of each group were treated OCT4-30Kc19 or/and BMP4. After two weeks of culture in the osteogenic medium, osteogenic capacity was analyzed. HUVECs treated with both OCT4-30Kc19 and BMP4 spheroids showed a significant increase in *COLI and OPN* osteogenic gene markers, by 3.10- and 2.92-folds, respectively (Fig. 5B). *OCN*, expressed in the late stage of osteogenesis, which is related to bone mineralization, also showed a significant increase by 5.32-folds. Although necrotic core was observed in HUVEC spheroid (Supplementary Fig. 3), it showed a well-preserved form even after two weeks of transdifferentiation, with a diameter of around 1,000 μm (Fig. 5C). High calcium deposition of HUVECs, treated with both OCT4-30Kc19 and BMP4, was shown by alizarin red s staining (Fig. 5D). Together, osteogenic gene expression profile and high calcium deposition capability confirm cell conversion into functional osteoblastic cells in vitro 3D cell spheroids*.*

### In vivo osteogenesis and angiogenesis via subcutaneous cell delivery after protein treatment

**Fig. 6:**
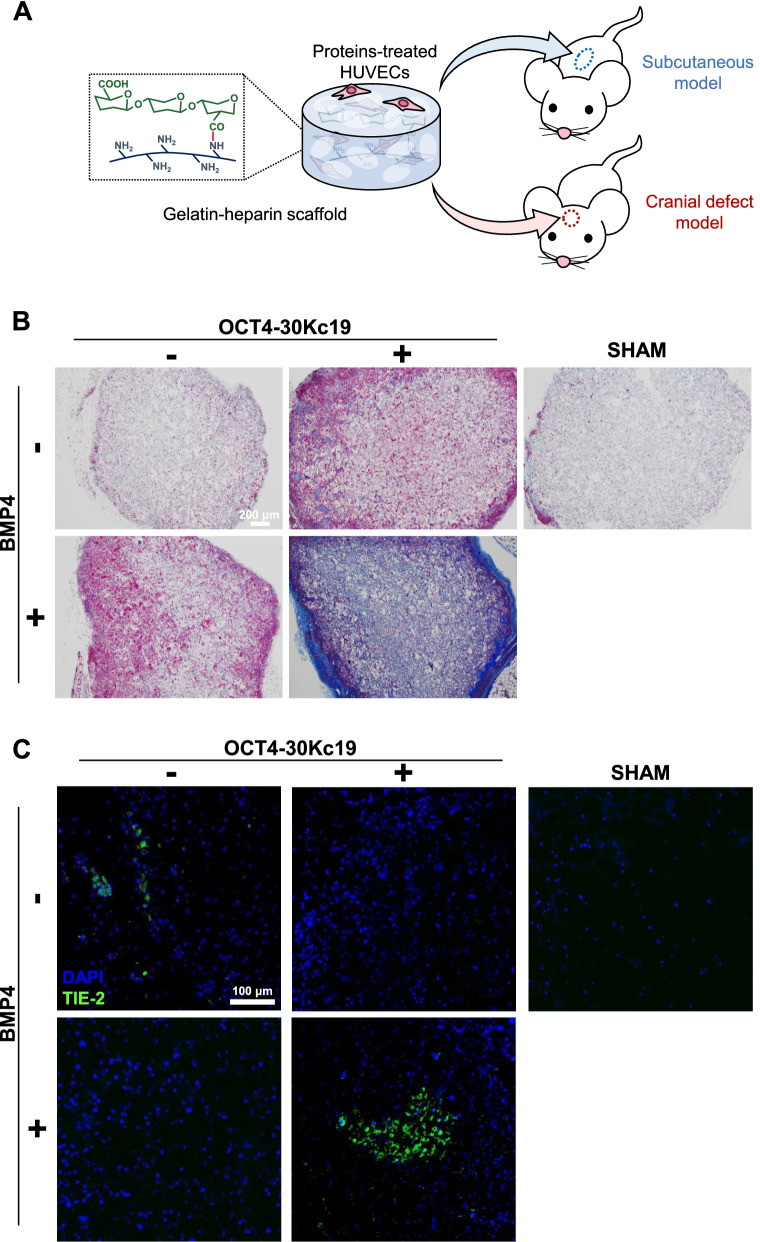
In vivo subcutaneous angiogenesis and osteogenic differentiation in HUVECs after OCT4-30Kc19 and BMP4 treatment. (**A**) Schematics of procedure of in vivo experiments, both for subcutaneous model and cranial defect model. HUVECs treated with OCT4-30Kc19 and BMP4 were seeded into gelatin-heparin scaffold. After cell-seeded scaffold were delivered into subcutaneous tissue, angiogenesis and osteogenic differentiation capacity was analyzed. Also, cell-seeded scaffolds were put in the defect area of sagittal crest of mice, bone regeneration capacity was analyzed. (**B**) Masson’s trichrome staining (MTC) of cell seeded gelatin-heparin scaffolds. Cells without treatment, only OCT4-30Kc19 treatment, only BMP4 treatment, both OCT4-30Kc19 and BMP4 treatment were each seeded on the scaffold and delivered into mouse subcutaneous tissue. After 6 weeks, MTC staining was performed. The sample treated with OCT4-30Kc19 and BMP4 showed the highest accumulation of collagen. Collagen is stained in blue. Scale bar, 200 μm. (**C**) Immunofluorescence staining of angiogenesis marker, TIE-2. TIE-2 was detected by anti-TIE 2-antibody (1:400). Both treatments of OCT4-30Kc19 and BMP4 on HUVECs elevated the expression of TIE-2, which indicates angiogenesis. Blue, green represent the nucleus and TIE-2 protein, respectively. Scale bar, 100 μm

Cells from each group were seeded on a gelatin-heparin scaffold used in previous experiments [25, 47]. To assess both the osteogenic and angiogenic potential in vivo, cell-seeded scaffolds were delivered into mouse subcutaneous tissue for 6 weeks (Fig. 6A). Scaffold samples collected after 6 weeks were analyzed for osteogenesis via MTC staining and angiogenesis via TIE-2 immunostaining (Fig. 6B and C). The sample group treated with OCT4-30Kc19 and BMP4 showed the highest collagen accumulation (stained in blue) suggesting the enhanced osteogenic capacity of cells (Fig. 6B). Like the trend shown in vitro, higher expression of an angiogenic marker, TIE-2, was observed in samples treated with OCT4-30Kc19 and BMP4 (Fig. 6C). Thus, cellular delivery into subcutaneous tissue shows both osteogenic and angiogenic characteristics of cells treated with OCT4-30Kc19 and BMP4 treatment.

### Bone regeneration in in vivo cranial defect mice model

**Fig. 7:**
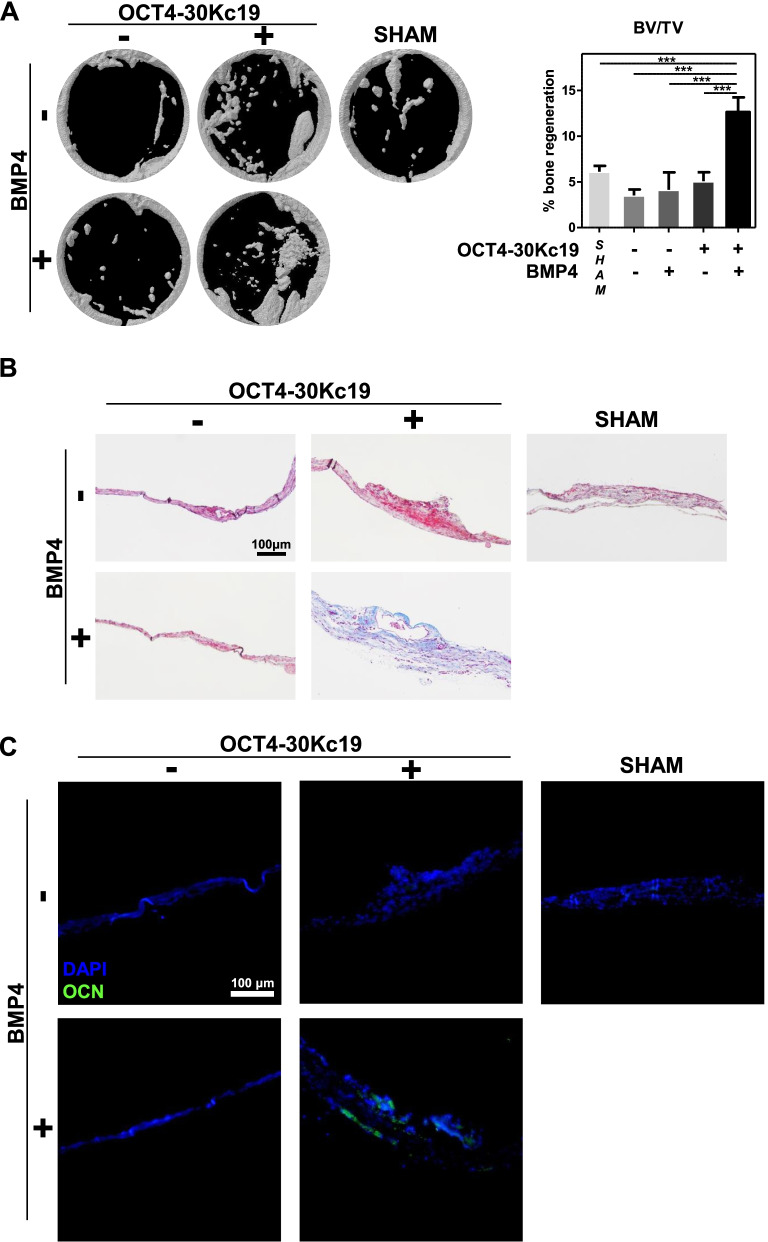
In vivo bone regeneration in cranial defect mice after 8 weeks. (**A**) Micro-computed tomography (micro-CT) 3D reconstruction image in cranial defects. Cranial defects with 4 mm diameter were made on mice and cell-seeded scaffolds were put in the defect area for 8 weeks. Before implantation, cells were treated with OCT4-30Kc19 or BMP4. The group with OCT4-30Kc19 and BMP4 treated cells had the highest bone regeneration potential. Quantitative analysis of bone volume to tissue volume (BV/TV) in each group (*n* = 3). ^***^p < 0.001. (**B**) Masson’s trichrome staining (MTC) of cranial defects. The sample treated with OCT4-30Kc19 and BMP4 showed the highest accumulation of collagen. Collagen is stained in blue. Scale bar, 100 μm. (**C**) Immunofluorescence staining of OCN. Expression of OCN in OCT4-30Kc19 and BMP4 samples was more prominent than in other groups. Anti-OCN-antibody (1:400) was used as the primary antibody for OCN detection. Scale bar, 100 μm

After confirmation of osteogenesis and angiogenesis via subcutaneous model, bone regeneration capacities of cells were examined. Cell-seeded scaffolds were put into the mouse cranial defect area and mice were sacrificed after 8 weeks (Fig. 6A). Micro-CT analysis of cranial bone demonstrated cells treated with OCT4-30Kc19 and BMP4 showed enhanced bone formation (Fig. 7A). Histological MTC staining displayed accumulation of collagen in OCT4-30Kc19 and BMP4 samples, consistent with the trend that appeared on tissue samples with subcutaneous cell injection (Fig. 7B). In addition, the thickness of the regenerated bone from OCT4-30Kc19 and BMP4 treated cells was higher than bone formation in other samples. Further confirmation of osteogenesis and the presence of human cells in the new bone was made with immunofluorescence staining with OCN (Fig. 7C). Expression of OCN in OCT4-30Kc19 and BMP4 sample was prominent compared to that of others suggesting human cell incorporation took place in new bone formation. Altogether, treatment of OCT4-30Kc19 and BMP4 in HUVECs resulted in efficient bone regeneration in vivo.

## Discussion

The application of transcription factor *OCT4* in iPSC-generation and transdifferentiation studies has shown its highly efficient cell reprogramming capability. A combination of *OCT4* with other transcription factors was used in cell reprogramming studies [20, 23, 49, 50]. In addition, *OCT4* alone induced iPSCs from neural stem cells, multilineage blood progenitors from dermal fibroblasts, and neural stem cells from cord blood cells [24, 26, 51]. Other studies have shown that endothelial cells that are genetically modified with *OCT4* result in increased pluripotency and enhanced angiogenic characteristics [52, 53]. In this study, we have shown treatment of OCT4-30Kc19 induced activation of *OCT4, SOX2,* and *NANOG* suggesting cells reached a pluripotent state.

Previous studies on 30Kc19 have not shown evidence of downstream gene activation from transcription factor delivery fused with 30Kc19. Fusion of 30Kc19 cell-penetrating protein allowed cell membrane penetration of OCT4 transcription factor. However, nucleus penetration is another essential step as a transcription factor to elicit change in genomic expressions. In this case, nuclear localization of OCT4 is necessary to bind and activate at the promoter regions of *SOX2, NANOG*, and *OCT4* itself. After penetration into the cell membrane via 30Kc19, innate nuclear localization sequence in OCT4 enabled delivery into the nucleus and this process was confirmed by SRM images and PCR analysis data. SRM images showed localization of OCT4-30Kc19 in the nuclei after 24 and 48 h of treatment (Fig. 2C). Elevation in fold induction in *SOX2, NANOG*, and *OCT4* suggested OCT4-30Kc19 protein that is delivered in the nucleus activated gene expressions (Fig. 2E). We confirmed that the fusion of OCT4 with 30Kc19 did not disrupt the original function of OCT4. The function as a transcription factor was maintained and OCT4 enhanced gene expressions of its downstream targets.

With the addition of the BMP4 growth factor, we were able to see the mesenchymal transition of HUVECs. A study by Medici et al*.* has shown endothelial-mesenchymal transition in HUVECs via BMP4 treatment promoting phosphorylation of activin receptor-like kinase 2 (ALK2) causing upregulation of SMAD pathway [31]. In 2011, Chen et al*.* was able to show BMPs, especially BMP4, can efficiently reprogram mouse fibroblasts into pluripotent states by triggering OCT4/SOX2 pathway [54]. The reprogramming capacity of BMP4 in combination with OCT4-30Kc19 was enhanced and resulted in osteogenic transdifferentiation and bone tissue formation.

Vascularization is an important part of bone tissue regeneration. The formation of the vascular network within fractured or defect areas offers delivery of nutrients and mesenchymal stromal cells that can also participate in the healing process. Thus, recent studies have focused on the importance of angiogenesis in bone tissue regeneration [55–57]. However, generating such complete bone tissue requires co-culturing more than one cell type such as endothelial cells, MSCs, and osteoblasts. Another approach will be combining materials that can stimulate angiogenesis or osteogenesis via conjugating appropriate growth factors to the substrate. In this study, our data from in vitro tube formation and gene expression profile of angiogenesis markers suggested angiogenic capacity in OCT4-30Kc19 and BMP4 treated cells and this was confirmed by in vivo subcutaneous delivery of OCT4-30Kc19 and BMP4 treated cells eliciting both osteogenic and angiogenic capabilities. In addition, evidence of the direct bone tissue formation from implanted cells in the cranial defect area was shown with micro CT images and staining with MTC and anti-human-OCN antibody. Thus, we provide complex bone tissue generated from angiogenesis and osteogenesis of a single cell source, HUVECs, without incorporating a co-culture system or VEGF-integrated materials.

For in vivo cell delivery into subcutaneous tissue and cranial defect area, cells were seeded onto a gelatin-heparin scaffold that has been previously used [25, 47]. The macroporosity of this scaffold has been extensively studied and its application in cell delivery was tested in previous experiments. Therefore, we applied the same scaffold in this study for in vivo delivery purposes. Its macroporous structure provides enough space for cells to attach and allows growth factors and nutrients to actively flow through. In addition, positively charged growth factors such as BMP2 and VEGF have an affinity towards negatively charged heparin [47]. In terms of cell cytotoxicity from the scaffold, previous studies confirmed that the gelatin-heparin scaffold did not affect the viability of the seeded cells [25, 47]. Cells seeded gelatin-heparin scaffold was implanted in the subcutaneous tissue to examine its angiogenesis ability, and cranial defect area to examine its osteogenesis ability.

In addition to the 2D in vitro and in vivo conditions, whether the protein can be delivered to the tissue-like cell aggregates was confirmed by the 3D spheroid model. Generation of the 3D spheroid model using MNPs have advantages in immediate and uniform cell gathering and high reproducibility. Also, because the MNPs are surrounded by a lipid bilayer, they are biocompatible materials without surface modification [58, 59]. And MNP-incorporating cells also can have an advantage when transplanted in vivo. They can be used in tracking cells or holding the transplanted cells at the desired position by external magnetic force [60–65].

Compared to the conventional 2D culture, 3D culture can have some changes in secretion of ECM, cell–cell interaction, gene expression levels [66–68]. Such trends were also shown in this experiment. Although the BMP4 and OCT4-30Kc19 treated group showed the most increased osteogenic differentiation capacity both in the 2D and 3D culture, OCT4-30Kc19 without BMP4 treated group showed different patterns in osteogenic marker gene expression levels in 2D or 3D culture (Figs. 4A and 5B). It can be inferred that additional factors from the 3D environment including limited oxygen and nutrients diffusion may affect the HUVECs’ behaviors and gene expression (Supplementary Fig. 3). From these different results in 2D and 3D culture, it can be seen that providing a more tissue-like 3D structure is an important factor in the generation of a complex bone tissue model. However, for the further long-term culture and modeling, it is necessary to solve internal necrosis caused by 3D formation [69, 70], which has been reported in several spheroid formation papers [68, 71–73]. In addition, unlike in the 2D culture, where the treated factors can reach the cells evenly, the surface of cell spheroids is mainly exposed to the treated factors. This shows that direct conversion is possible with the cell-penetrating OCT4-30Kc19 and BMP4 factors even at the tissue level. So, it opens the possibility of cell therapy that can transform the whole tissue into desired cell types. Direct conversion at the tissue level could alleviate impaired balances between adipogenesis and osteogenesis occurred in osteoporosis [74–76], by targeting increased portion of adipose tissue in the bone marrow.

In this paper, we have successfully demonstrated a transdifferentiation strategy that does not provoke safety risks that arise from using viral, bacterial plasmid deliveries or treating chemicals. Current issues in stem cell and cell-based therapy involve the rise of unintended genomic changes or tumorigenesis from using iPSCs or declined multipotency in aged MSCs causing challenges in using MSCs as a cell source. MSCs are abundant and can be isolated from many different parts of the body including dental pulp, bone marrow, umbilical cord, adipose tissues, etc. [77–80]. However, the differentiation capacity of MSCs declines with aging resulting in difficulties when used as an autologous cell source in older patients who are most likely to suffer from bone-related diseases [81, 82]. In addition to the declining self-renewal, multipotent state, the number of MSCs significantly reduces with donors’ ages [83]. Our method of transdifferentiation both encompasses these problems in cell-based therapy by bypassing direct genomic alterations with 30Kc19 protein and using HUVECs as a cell source.

## Conclusion

In this study, we demonstrated that the treatment of OCT4 transcription factor into cells via fusion with cell-penetrating protein, 30Kc19, showed successful penetration into cell membrane as well as cell nuclei with relevant molecular changes. Coupled with the BMP4 treatment, cellular transdifferentiation can be induced with both osteogenic and angiogenic potential, which were proven in vitro*,* in 3D cell spheroids, and in vivo cellular delivery. This study suggests an expanded application of 30Kc19 cell penetrating protein in transdifferentiation studies and implicates future application to bone-related disorders.

## Supplementary Information

**Additional file 1:** **Supplementary Fig. 1**. Cytotoxicity of OCT4-30Kc19 protein on HUVECs. Quantitative analysis of live cells. After treating HUVECs with different concentrations of OCT4-30Kc19 protein for 24 hrs, cells were stained by live and dead kit and live cell percentage was measured (*n*= 3). ***p < 0.001. **Supplementary Fig. 2**. Cytotoxicity of MNPs on HUVECs. After incubating HUVECs with various concentrations of MNPs for 1, 3, and 7 days, viability of each group was quantified by CCK assay (*n* = 4). **p < 0.01, ***p <0.001. **Supplementary Fig. 3**. Viability of OCT4-30Kc19 and BMP4-treated HUVEC spheroid. Live and dead assay for HUVEC spheroid. The live HUVECs were shown as green and the dead HUVECs were shown as red. Scale bar, 200 µm.

## Data Availability

All data generated or analyzed during this study are included in this published article.

## Abbreviations

MSC: Mesenchymal stem cell
HIV: Human immunodeficiency virus
HUVECs: Human umbilical vein endothelial cells
iPSCs: Induced pluripotent stem cells
OCT4: Octamer-binding transcription factor 4
BMP4: Bone morphogenetic protein 4
RUNX2: Runt-related transcription factor 2
ALP: Alkaline phosphatase
OCN: Osteocalcin
TAT: Trans-activator of transcription
SOX2: SRY-box transcription factor 2
KLF4: Kruppel-like factor 4
VEGF: Vascular endothelial growth factor
αSMA: Alpha smooth muscle actin
VECAD: VE-cadherin
VEGFR-2: Vascular endothelial growth factor receptor-2
ALK2: Activin receptor-like kinase 2
BSP: Bone sialoprotein
OPN: Osteopontin
COL I: Collagen type I
VP22: Virus protein 22
ARS: Alizarin red s staining
MTC: Masson’s trichrome stain
FPLC: Fast protein liquid chromatography
EGM-2: Endothelial cell growth basal medium-2
SDS-PAGE: Sulfate–polyacrylamide
PVDF: Polyvinylidene difluoride
BSA: Bovine serum albumin
FBS: Fetal bovine serum
TBS: Tris-buffered saline
MNPs: Magnetic nanoparticles
PBS: Phosphate-buffered saline
NGS: Normal goat serum
PFA: Paraformaldehyde
DW: Deionized water
PCR: Polymerase chain reaction
cDNA: Complementary DNA
EDC: 1- Ethyl-3-(3-(dimethylamino)propyl)carbodiimide
NHS: Sulfohydroxysuccinimide
EndMT: Endothelial-mesenchymal transition
SD: Standard deviation
SRM: Super resolution microscope
O.D: Optical density
MFI: Mean fluorescence intensity
BV/TV: Bone volume/tissue volume
