# Supplementary material for: Cellular direct conversion by cell penetrable OCT4-30Kc19 protein and BMP4 growth factor
Source: Biomater Res. 2022 Jul 14;26:33. doi: 10.1186/s40824-022-00280-8 (PMC9281139; doi:10.1186/s40824-022-00280-8)
Supplement: Supplementary file 1 — Additional file 1: Supplementary Fig. 1. Cytotoxicity of OCT4-30Kc19 protein on HUVECs. Quantitative analysis of live cells. After treating HUVECs with different concentrations of OCT4-30Kc19 protein for 24 hrs, cells were stained by live and dead kit and live cell percentage was measured (n= 3). ***p < 0.001. Supplementary Fig. 2. Cytotoxicity of MNPs on HUVECs. After incubating HUVECs with various concentrations of MNPs for 1, 3, and 7 days, viability of each group was quantified by CCK assay (n = 4). **p < 0.01, ***p <0.001. Supplementary Fig. 3. Viability of OCT4-30Kc19 and BMP4-treated HUVEC spheroid. Live and dead assay for HUVEC spheroid. The live HUVECs were shown as green and the dead HUVECs were shown as red. Scale bar, 200 µm. [file 40824_2022_280_MOESM1_ESM.pdf]

Supporting Information

Cellular direct conversion by cell penetrable OCT4-30Kc19 protein and BMP4 growth factor

Seung Hyun L. Kim[1†], Sungwoo Cho[2†], Seoyeon Kim[2†], Janet Kwon[1,6], Jaeyoung Lee[5], Rachel H. Koh[3], Ju Hyun Park[5], Hwajin Lee[4*], Tai Hyun Park[1,2,3*], and Nathaniel S. Hwang[1,2,3*]

[1]Interdisciplinary Program in Bioengineering, Seoul National University, Seoul, 08826, Republic of Korea

[2]School of Chemical and Biological Engineering, Institute of Chemical Processes, Seoul National University, Seoul, 08826, Republic of Korea

[3]Bio-Max/N-Bio Institute, Institute of Bioengineering, Seoul National University, Seoul, 08826, Republic of Korea

[4]School of Dentistry, Seoul National University, Seoul, 08826, Republic of Korea

[5]Department of Biomedical Science, Kangwon National University, Chuncheon, Gangwon-do, 24321, Republic of Korea

[6]Department of Biomedical Engineering, University of California, Davis, California, 95616, United States of America

*corresponding authors

nshwang@snu.ac.kr

thpark@snu.ac.kr

hwajin2k@gmail.com

†These authors contributed equally to this work.

Supplementary Figures

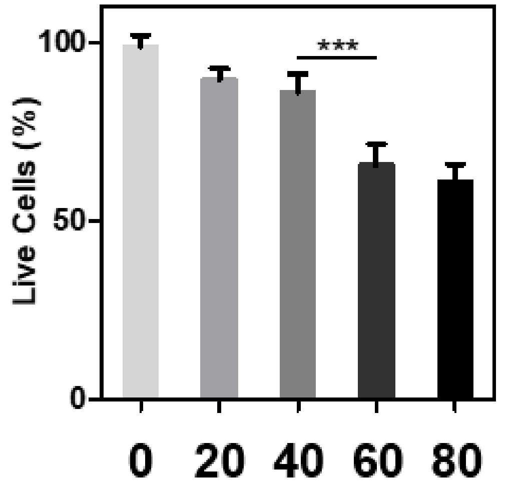

Supplementary Figure 1. Cytotoxicity of OCT4-30Kc19 protein on HUVECs.

Quantitative analysis of live cells. After treating HUVECs with different concentrations of OCT4-30Kc19 protein for 24 hrs, cells were stained by live and dead kit and live cell percentage was measured (n = 3). $^{***}p < 0.001$.

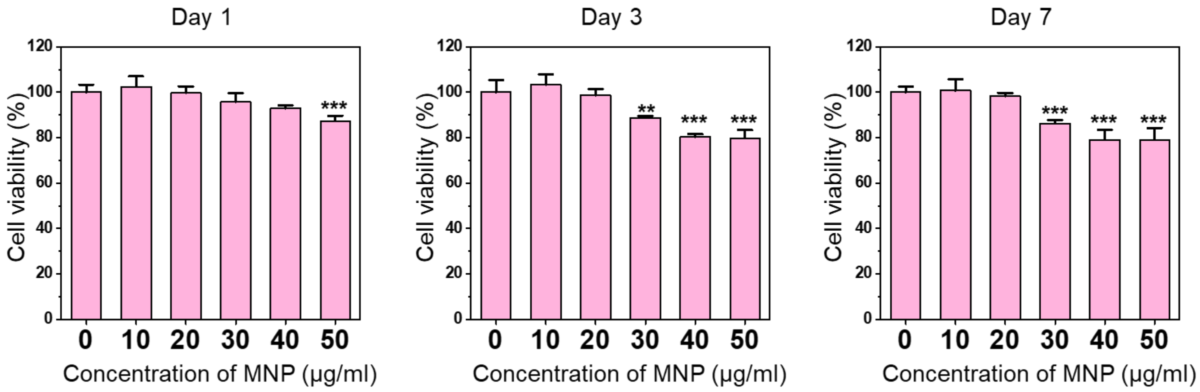

Supplementary Figure 2. Cytotoxicity of MNPs on HUVECs.

After incubating HUVECs with various concentrations of MNPs for 1, 3, and 7 days, viability of each group was quantified by CCK assay (n = 4). $^{**}p < 0.01$, $^{***}p < 0.001$.

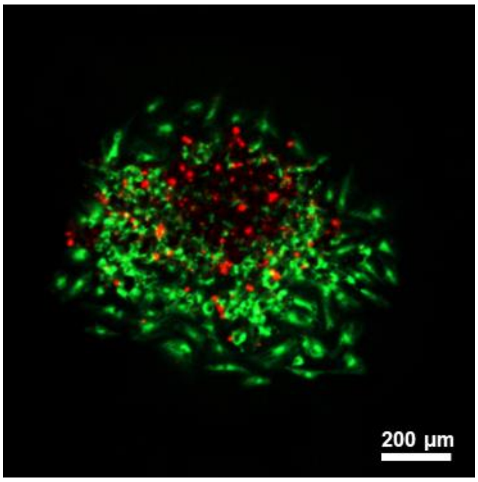

Supplementary Figure 3. Viability of OCT4-30Kc19 and BMP4-treated HUVEC spheroid.

Live and dead assay for HUVEC spheroid. The live HUVECs were shown as green and the dead HUVECs were shown as red. Scale bar, 200 µm.
